# *Lactobacillus* Endocarditis-Associated Glomerulonephritis Complicated by anti-Coagulant Nephropathy and Renal Amyloidosis

**DOI:** 10.1155/2019/6198380

**Published:** 2019-12-17

**Authors:** Mitchell Moosavi, Jonathan E. Zuckerman

**Affiliations:** Department of Pathology and Laboratory Medicine, University of California, Los Angeles, Los Angeles, CA, USA

## Abstract

Kidney injury is a well-known sequelae of infectious endocarditis. Various types of kidney injury can be seen, including endocarditis-associated glomerulonephritis, and may affect nearly half of the patients with infectious endocarditis. *Lactobacillus* species are an infrequently documented cause of endocarditis. We present a case of *Lactobacillus* endocarditis-associated glomerulonephritis in a patient with a complex medical history including *Lactobacillus* infection of an artificial heart valve. To our knowledge, this is the first reported case of development of endocarditis-associated glomerulonephritis secondary to *Lactobacillus* species organisms. Furthermore, the patient's renal biopsy revealed several frequently overlooked concomitant findings including anti-coagulant nephropathy and renal amyloidosis.

## 1. Clinical Presentation

The patient is a 72 year old man with diabetes mellitus, stage II chronic kidney disease, coronary artery disease, and mechanical heart valve complicated by a *Lactobacillus* infection, for which he was receiving antibiotics. The patient presented with acute kidney injury and a serum creatinine of 3.6 mg/dL as well as supratherapeutic warfarin level (international normalized ratio (INR) of 6.0). Serologic evaluation was significant for positive Antistreptolysin O antibody titer. Autoimmune serologic work up was negative. The clinical differential diagnosis included acute tubular injury, acute glomerulonephritis, and thrombotic microangiopathy.

## 2. Kidney Biopsy (Figure 1)

By light microscopy, glomeruli exhibited a membranoproliferative pattern of injury including double contour formation, segmental endocapillary hypercellularity, and prominent fuchsinophilic capillary loop deposits as well as mesangial hypercellularity. Two glomeruli exhibited fibrous crescents. There was diffuse tubular injury accompanied by luminal red blood cell casts and fresh blood, to a degree out of proportion to the glomerular injury. The interstitium was variably edematous and infiltrated by inflammatory cells including lymphocytes, plasma cells, and scattered eosinophils associated with mild tubulitis. There was also scattered amorphous eosinophilic deposits present within interstitial spaces which showed apple-green birefringence under polarized light when stained with congo red. There was moderate cortical scarring. Arterial and arteriolar sclerosis without vasculitis or thromboses. Immunofluorescence microscopy demonstrated diffused global granular glomerular capillary wall and mesangial region staining with IgG (2+), IgA (2-3+), IgM (3-4+), C1q (3-4+), C3 (4+), and Kappa (2-3+), and Lambda (2+) light chains. Ultrastructural studies demonstrated many finely granular electron dense deposits in mesangial and subendothelial locations. Subendothelial spaces were widened with interposition of subendothelial deposits, cell processes, and neomembrane. There were no tubuloreticular inclusion or extra glomerular deposits.

## 3. Diagnosis

Infection-related glomerulonephritis secondary to *Lactobacillus* endocarditis with active and chronic components with superimposed anticoagulant-associated nephropathy and interstitial amyloidosis. Additionally, a chronic active tubulointerstitial nephritis was present which was favored to represent either a component of the glomerulonephritis or more likely a concomitant allergy induced process secondary to the antibiotic therapy.

The etiology of the acute kidney injury was considered multi-factorial with contribution from the glomerulonephritis, anticoagulant-associated nephropathy, and interstitial nephritis. The amyloidosis was favored to be an incidental finding. The amyloid deposits did not stain for either light chain or serum amyloid A on immunofluorescence and immunohistochemistry respectively. Unfortunately, there was insufficient residual tissue to perform mass-spectrometry characterization. Thus the type of amyloidosis in this case could not be determined.

## 4. Discussion

Glomerulonephritis is to be seen in up to 40–50% of patients with infectious endocarditis [1]. The manifestations of renal involvement are variable [2] and can include hematuria, proteinuria, infarction related to septic emboli, damage secondary to deposition of immune complexes, direct immune mediated destruction, and secondary interstitial nephritis from antibiotic and drug treatment [1, 2]. Common pathogenic agents for infectious-endocarditis associated glomerulonephritis include Gram-positive cocci; however, the etiologic agents are diverse [1, 2]. The pathogenesis of endocarditis-associated glomerulonephritis is thought to involve immunologic injury. The finding of circulating immune complexes and subendothelial deposits in patients with endocarditis is supportive of this mechanism [1, 2].

Infectious-endocarditis associated glomerulonephritis can manifest in a number of distinct patterns, including focal and diffuse forms of crescentic and/or proliferative glomerulonephritis [1, 2]. Immune complex deposition is variable and may show a pauci-immune pattern [1]. When present the immune complexes generally show staining for IgG and C3 deposits; however, IgM-dominant, IgA-dominant or complete “full house” staining can be seen [1]. Sub-epithelial humps may be seen on ultrastructural examination [1]. Amyloidosis is a rare complication seen in bacterial endocarditis and other chronic infections [6]. It has been implicated as a cause of renal dysfunction in a number of patients with endocarditis due to deposition within glomeruli. The amyloid deposits are most commonly of the light chain (AL) or inflammatory type (AA) [7, 8]. The co-presence of warfarin-related nephropathy in patients with histories of bacterial endocarditis and endocarditis-associated glomerulonephritis have been documented [9, 10].

*Lactobacillus* infectious endocarditis is a rare cause of bacterial endocarditis [3, 4] with a mortality rate as high as 30–48% [3]. Although considered commensal flora of the gastrointestinal tract, female genitourinary tract and oropharynx, this facultative Gram positive rod-shaped bacterium has been implicated in a vast number of infections ranging from dental caries to intra-abdominal abscesses to endocarditis [3, 4]. Risk factors for *Lactobacillus* bacteremia include impaired immunity, extensive use of antibiotics, diabetes mellitus, structural heart disease as well as the consumption of probiotics in select cases [3–5]. A limitation of this study is the difficulty in culturing certain strains of Streptococci and with the positive Antistreptolysin O antibody titer, these species of Streptococci remain possible etiologic agents [11]. Nevertheless, a number of *Lactobacillus* subspecies have been reported in the literature as the etiologic agents of endocarditis [5]. The current case presentation is the first report (to the author's knowledge) of *Lactobacillus* endocarditis associated glomerulonephritis.

## Figures and Tables

**Figure 1:**
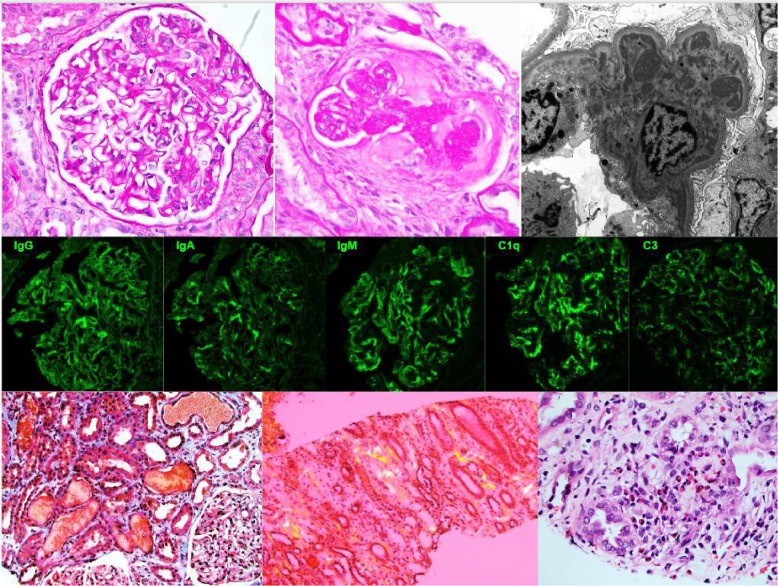
Renal biopsy findings. Membranoproliferative glomerulonephritis showing (top row-left) segmental endocapillary hypercellularity and double contour formation (top-row-middle) old fibrous crescent by light microscopy (periodic acid schiff stain) and (top-row-right) mesangial and subendothelial immune complex deposits by electron microscopy. (Middle-row) representative micrographs of the immunofluorescence studies. Additional tubulointerstitial findings included (bottom-row-left) numerous occlusive red blood cell casts in the tubules (trichrome stain) (bottom-row-middle) interstitial amyloidosis showing apple-green birefringence on congo red stain (bottom-row-right) numerous interstitial eosinophils suggestive of allergic/drug-induced acute interstitial nephritis (H&E stain).
